# Structured reporting for fibrosing lung disease: a model shared by radiologist and pulmonologist

**DOI:** 10.1007/s11547-017-0835-6

**Published:** 2017-12-11

**Authors:** Nicola Sverzellati, Anna Odone, Mario Silva, Roberta Polverosi, Carlo Florio, Luciano Cardinale, Giancarlo Cortese, Giancarlo Addonisio, Maurizio Zompatori, Giorgia Dalpiaz, Sara Piciucchi, Anna Rita Larici, The Italian Structured Report on Fibrosing Lung Disease Consort, Carlo Agostini, Carlo Albera, Domenico Attinà, Giuseppe Battista, Elena Bertelli, Giuseppina Bertorelli, Claudio Bnà, Martina Bonifazi, Lorenzo Bonomo, Andrea Borghesi, Lucio Calandriello, Antonella Caminati, Diana Capannelli, Stefania Cerri, Federica Ciccarese, Davide Colombi, Marco Confalonieri, Annaemilia Del Ciello, Giovanni Della Casa, Roberto Dore, Fabio Falaschi, Alessandra Farchione, Beatrice Feragalli, Paola Franchi, Giampaolo Gavelli, Sergio Harari, Fabrizio Luppi, Fabio Maggi, Maria Antonietta Mazzei, Manuela Mereu, Gianluca Milanese, Stefano Palmucci, Rosa Lucia Patea, Alberto Pesci, Marco Piolanti, Venerino Poletti, Gaetano Rea, Luca Richeldi, Paola Rogliani, Chiara Romei, Paola Rottoli, Alessandro Sanduzzi-Zamparelli, Alfredo Sebastiani, Gianluigi Sergiacomi, Gian Alberto Soardi, Lucia Spaggiari, Paolo Spagnolo, Sara Tomassetti, Rocco Trisolini, Adele Valentini, Carlo Vancheri, Valentina Vespro, Luca Volterrani

**Affiliations:** 1 Section of Radiology, Unit of Surgical Sciences, Department of Medicine and Surgery (DiMeC), University of Parma, Parma, Italy; 2 Department of Medicine and Surgery (DiMeC), University of Parma, Parma, Italy; 3 Department of Radiology, Ospedali di San Donà di Piave e Jesolo, Padua, Italy; 4 Department of Radiology, Istituto Tumori “Giovanni Paolo II” di Bari, Bari, Italy; 5 Department of Radiology, S. Luigi Gonzaga Orbassano, Turin, Italy; 6 Department of Radiology, Ospedale Maria Vittoria, Turin, Italy; 7 Radiology, Policlinico S. Orsola-Malpighi, University of Bologna, Bologna, Italy; 8 Department of Radiology, Azienda Unità Sanitaria Locale di Bologna, Bologna, Italy; 9 Department of Radiology, GB Morgagni Hospital, Forlì, Italy; 10 Department of Radiological Sciences, Institute of Radiology, Università Cattolica del Sacro Cuore, Rome, Italy

**Keywords:** Structured report, Standardized report, Consensus, Lung fibrosis, High-resolution computed tomography

## Abstract

**Objectives:**

To apply the Delphi exercise with iterative involvement of radiologists and pulmonologists with the aim of defining a structured reporting template for high-resolution computed tomography (HRCT) of patients with fibrosing lung disease (FLD).

**Methods:**

The writing committee selected the HRCT criteria—the Delphi items—for rating from both radiology panelists (RP) and pulmonology panelists (PP). The Delphi items were first rated by RPs as “essential”, “optional”, or “not relevant”. The items rated “essential” by < 80% of the RP were selected for the PP rating. The format of reporting was rated by both RP and PP.

**Results:**

A total of 42 RPs and 12 PPs participated to the survey. In both Delphi round 1 and 2, 10/27 (37.7%) items were rated “essential” by more than 80% of RP. The remaining 17/27 (63.3%) items were rated by the PP in round 3, with 2/17 items (11.7%) rated “essential” by the PP. PP proposed additional items for conclusion domain, which were rated by RPs in the fourth round. Poor consensus was observed for the format of reporting.

**Conclusions:**

This study provides a template for structured report of FLD that features essential items as agreed by expert thoracic radiologists and pulmonologists.

**Electronic supplementary material:**

The online version of this article (10.1007/s11547-017-0835-6) contains supplementary material, which is available to authorized users.

## Introduction

The radiology report is an essential part of the service that radiologists provide to both patients and referring physicians, in any field of medicine. It records information for future use and it is part of the legal records for the episode of care [1]. The radiology report’s structure and content may vary according to several factors, including the clinical inquiry, and the radiologist’s expertise and education.

Free text reporting is still the most common format in clinical radiology. However, free text clinical reports may heterogeneously render the core information (e.g., language and cultural variability), making it difficult to compare reports or find specific details [2, 3]. This is particularly true for diffuse lung disease (DLD), which is often a challenging diagnosis and prone to variable description by high-resolution computed tomography (HRCT). Notably, there is no consensus on the relevant findings that should serve as core mandatory information to the referring physician. For instance, reporting enlarged lymph-nodes might result misleading in patients with fibrosing lung disease (FLD) because these abnormalities frequently coexist without specific clinical implication.

A number of initiatives are being promoted by the major international societies of radiology to disseminate the use of structured reporting [4]. Potentially, the use of structured reporting may improve consistency in clinical radiology [5]. It might reduce the rate of overlooking important findings as well as improve communication with referring physician. Several studies have explored pros and cons of the structured reporting in various settings. Some of them showed some benefit whereas others did not [2, 5–7]. However, it seems that most radiologists are encouraging and appreciating the use of structured reporting, especially in among subspecialist radiologists [8].

To our knowledge, there is no proposed structured reporting template for HRCT scans of subjects with DLD to guide radiologists in the systematic reporting in the framework of findings and synthesize a final clinical hypothesis. Following debates between expert pulmonologists and chest radiologists at the Italian national meetings national meetings, respectively, from the Italian societies of respiratory medicine and the Italian society of medical radiology (SIRM), we hypothesized that a structured report might be particularly helpful for HRCT of patients with FLD.

Within the reference standard of multidisciplinary discussion, the radiology report remains the primary method of communication between radiologists and clinicians, particularly in non-referral centers. A simple stepwise approach can aid HRCT interpretation and is especially applicable to FLD, thus fitting structured reporting [9]. In fact, reducing the chance of omitting important findings (e.g., traction bronchiectasis or pulmonary emphysema), or supplying an interpretation of the radiological pattern, would be paramount to guide the referring physician in critical clinical decisions for patients with FLD.

This study was undertaken to enact critical shared discussion between chest radiologists and pulmonologists by means of multi-round consensus-building Delphi exercise, to develop a comprehensive focused structured reporting template for HRCT of patients with FLD. The study objectives were to develop a list of HRCT criteria to describe FLD, to learn the most relevant parameters according to the point of view of pulmonologist, and to assess the agreement among experts on the proposed criteria.

## Materials and methods

A six members writing committee proposed the HRCT criteria and parameters—the Delphi items –to the panelists rating. The writing committee members had at least 10 years of experience and authored at least 10 studies in imaging of DLD and they did not participate in the following Delphi survey. The list of Delphi items was based on the evidence from the literature in FLD and the experience of each writing committee member.

### Selection of the Delphi domains and items

A literature search was performed in Medline to identify publications relevant to the HRCT features of FLDs from 2002 idiopathic interstitial pneumonia classification document to January 2016 [10]. The full text of the selected studies was reviewed by two out of six members of the writing committee, who developed and shared the initial list of Delphi items with the other writing committee members via emails and teleconferences.

The structured report was divided into three domains according to the American College of Radiology handbook for residents: (a) initial considerations, (b) HRCT findings, (c) Conclusions [11]. The Delphi items proposed by the writing committee are detailed in Table 1 according to domain belonging.

**Table 1:** List of Delphi items proposed by the writing committee for panelists survey

Initial considerations	HRCT findings	Conclusions
Available clinical indication	Comparison of CT findings with prior scan, indicating change of each CT finding	CT pattern: (1) DEFINITE UIP (2) POSSIBLE UIP (3) ACUTE COMPLICATIONS IN UIP (4) NSIP (5) NSIP-OP (6) SARCOIDOSIS (7) PPFE
CT protocol details	Initial assessment of signs of lung fibrosis (honeycombing, traction bronchiectasis, signs of volume loss)	Proposal for the subsequent diagnostic test
Comparison with prior CT examinations	Confidence on honeycombing against traction bronchiectasis	Indication for the timing of CT follow-up
Differences in CT technique with prior examinations	Avoid description of absent findings	
Motion artifacts	Description of all CT findings	
	Description of the most relevant CT findings only (e.g., honeycombing, traction bronchiectasis, signs of volume loss)	
	Disease distribution on both axial and cranio-caudal planes	
	Differentiation between macro- and micro-cystic honeycombing	
	Description of the reticular opacities subtypes (e.g., intralobular or interlobular)	
	Emphysema subtype, including the so-called airspace enlargement with fibrosis	
	Quantitation of FLD extent as percentage of the lung volume	
	Quantitation of FLD extent according to three categories of severity	
	Quantitation of FLD extent for any disease OR for sarcoidosis and systemic sclerosis only	
	Quantitation of emphysema extent as percentage of the lung volume	
	Quantitation of emphysema extent according to three categories of severity	
	Report air trapping only when expiratory CT scan is performed	
	Suggest air trapping also on inspiratory CT scan	
	Report enlarged pulmonary artery for any disease OR for sarcoidosis and systemic sclerosis only	
	Report enlarged lymph-nodes	

*OP* organizing pneumonia; *PPFE* Pleuro-Parenchymal Fibroelastosis

The domain “Initial considerations” included information needed for pre-test description of the clinical scenario and the quality of HRCT, as follows: available clinical indication, HRCT technique details (including technical differences potentially affecting the study comparison), and disclosure of motion artifacts.

The domain “HRCT findings” included the comparison of the HRCT findings with prior examinations, notably referring to extent and type of radiological findings (e.g., reticular opacities are increased in extent, traction bronchiectasis look more severe, ground-glass opacity resolved, ground-glass opacity evolved towards overt reticulation, etc.). Furthermore, hierarchy of HRCT findings description was investigated with particular emphasis on the key components of the FLD, namely honeycombing, traction bronchiectasis, and signs of volume loss [9]. Furthermore, it was asked whether the description of the HRCT findings had to be concise or include also missing finding (e.g., not signs of honeycombing, not air trapping, etc.). It was also investigated the relevance of disclosure of the confidence in diagnosing and differentiating honeycombing and traction bronchiectasis, which is a critical task even among expert chest radiologists [12].

Quantitative information was also among the items of “HRCT findings”, namely the relevance of reporting the extent of fibrosis and emphysema in the structured report. Furthermore, the format of preferred visual scoring was proposed as either continuous variable (e.g., percentage) or as category (e.g., mild, moderate, or severe). Of note, no cut-off values were a priori set to define the categories of disease extent (e.g., the category should be assigned according to radiologist subjective impression). In addition, it was investigated whether extent should be provided for any FLD or only for individual disorders (e.g., sarcoidosis, systemic sclerosis) for which literature supported the prognostic value of such data at the time of the present study [13, 14]. Scientific references were supplied to the panelists for informed review of the aforementioned items.

The domain “Conclusions” was meant to investigate the relevance of reporting the HRCT pattern (e.g., usual interstitial pneumonia, UIP; non-specific interstitial pneumonia, NSIP, etc.), of proposing further diagnostic evaluation, and of indicating the timing for HRCT follow-up.

### Selection of the study panelists

The Italian Chest Imaging Subspecialty Society indicated a list of 53 expert radiologists (38 males, age range 30–72 years, median age 43 years, median years of experience in DLD 11 years), all active members of the Section of Thoracic Imaging of the SIRM, to participate in the Delphi survey (i.e., the radiology panelists—RPs). Selection criteria for RPs were as follows: (a) ≥ 5 years of experience in imaging of DLD (as reported in the society database); (b) authorship of ≥ 3 articles on DLD in peer-reviewed journal. Likewise, 18 pulmonologists (13 males, age range 38-61 years, median age 53 years, median years of experience in DLD 15) panelist (PP) were selected according to the following selection criteria: (a) ≥ 10 years of experience in DLD; (b) authorship of ≥ 10 articles on DLD in peer-reviewed journal. The selected RPs and PPs were contacted by email, informed about the aims and methodology of the study.

### Overview of the Delphi exercise

The Delphi exercise was run by a biostatistician (with 5 years of experience), through emails exchange.

First, RPs were asked to classify Delphi items into three categories, as follows: (a) “essential”; (b) “optional”; or (c) “not relevant” for the HRCT structured reporting. Items rated “essential” were further classified according to the format of reporting into “free text” or “outlined” (i.e., by a fixed check-list of descriptors or categories). A threshold of 80% “essential” rating by the RPs was set to retain items in the final structured report. Thereafter, items rated “essential” by less than 80% of the RPs were subsequently used to compile the Delphi item list for the PPs round. Again, a threshold of 80% “essential” rating by the PPs was set to retain items in the definite structured report. Items rated “not relevant” were considered useless or misleading, therefore, excluded from the final structured report. Furthermore, all panelists were allowed to suggest additional items to be included in the item list for the subsequent round; this fostered the open discussion that this inter-specialty consultation was meant for.

**Fig. 1:**
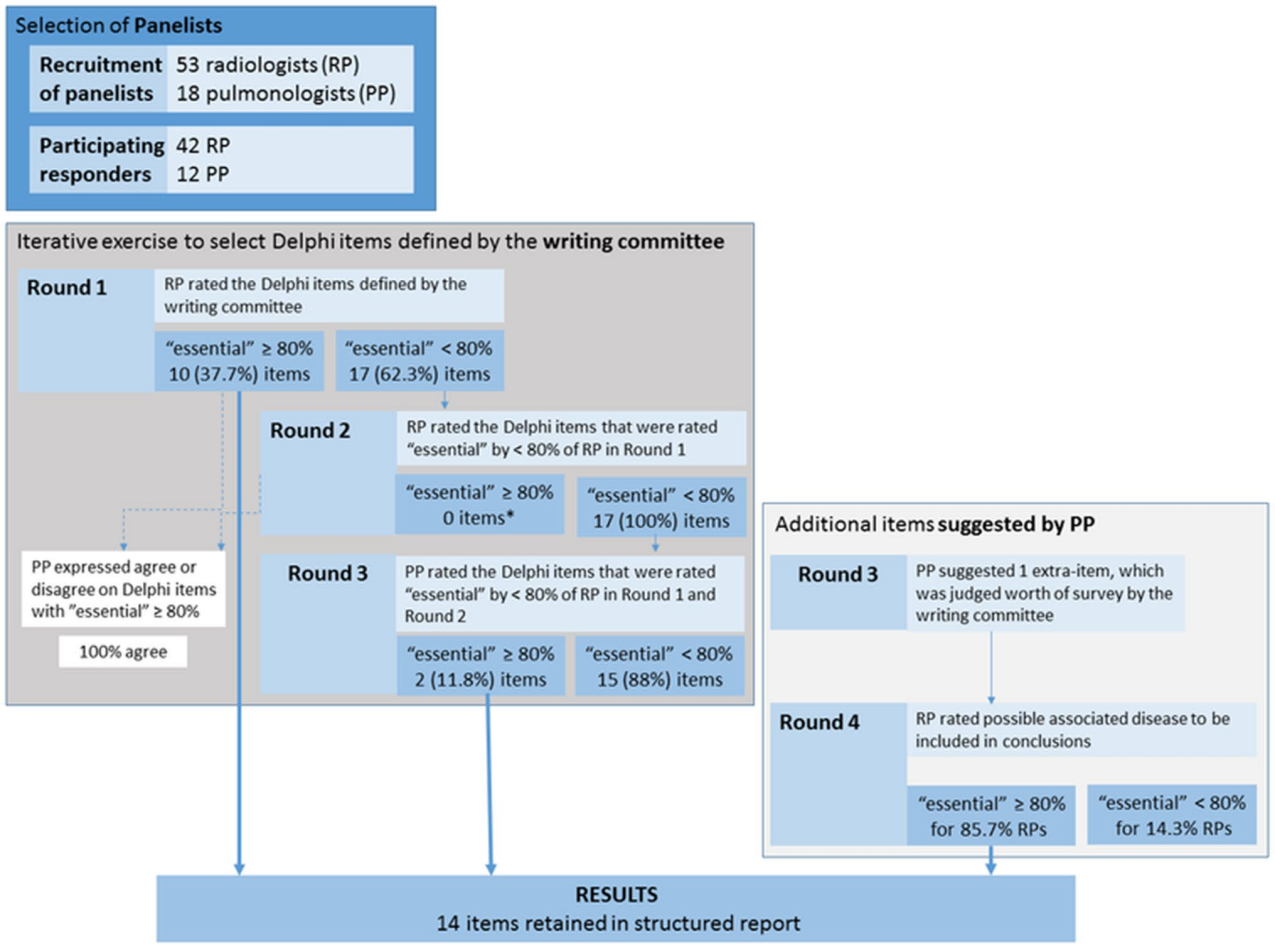
Diagram summarizing the Delphi rounds results

In addition, PPs were also asked to judge the Delphi items that had been rated “essential” by the RPs, as follows: agree or disagree. The detailed sequence of steps of the Delphi survey is shown in Fig. 1.

### Data analysis

Survey analysis was prospectively performed round by round [15]. In particular, the results of round 1 provided the content for sequential surveys. The cut-off value to retain an item within the exercise and eventually in the HRCT reporting model was ≥ 80% “essential” rating [16, 17]. Items with < 80% “essential” rating were iteratively rated at subsequent rounds. Likewise, this cut-off value was also used to select the most appropriate format of reporting of each retained item (e.g., to be given as free text or to be outlined).

## Results

Four Delphi rounds were performed to obtain the final structured report. A total of 42/53 (79.2%) invited radiologists agreed to participate as RPs. All of them completed the first round, 41 (97.6%) and 36 (85.7%) completed the second and the fourth round, respectively. A total of 12/18 (66.7%) pulmonologists agreed to participate as PPs and completed one Delphi round (i.e., the third round).

The result of each round is given in supplementary Tables 1–4 and summarized in Fig. 1. In round 1, 10/27 (37.7%) Delphi items were rated “essential” by more than 80% of RPs. In particular, total or nearly total agreement was reached for the following items: presence of prior HRCT scans for comparison, initial description of the variation of HRCT findings as compared to prior scans, and assessment of the disease distribution. No format option reached the cut-off of 80% and was re-rated in round 2 by the RPs. A number of panelists expressed no preference for the format of reporting as outlined in supplementary Tables.

In round 2, 17/27 (63%) items were re-rated by the RPs. A 80% consensus was not reached for any of them. A consensus greater than 80% was recorded upon the outline format for four out of 10 (40%) items that had been judged “essential” in round 1. Ten out of 42 (24%) RPs suggested to add an item for ancillary findings (e.g., pleural plaques, dilated esophagus, etc.), and report it as “free text”.

In round 3, PPs rated “essential” the extent of the visual scoring of both FLD (expressed as percentage lung volume involvement) and emphysema (expressed as categories). No sufficient consensus was reached for the remaining 15 items that had not been rated “essential” by the RPs. Besides, the PPs unanimously agreed on both the set of 10 “essential” items chosen by the RPs. Furthermore, three (25%) PPs suggested to expand on the conclusions domain by including a more detailed list of HRCT patterns and potential association or cause (e.g., unknown connective tissue disease, asbestosis, etc.) of FLD predictable from the HRCT findings. This was regarded as worth of rating by the radiologists writing committee that agreed upon a list of both HRCT patterns and associations of FLD.

In round 4, RPs chose between including only the HRCT patterns list or also suggesting any association or cause of the FLD in the structured report. There was substantial agreement upon suggesting the association or cause of the FLD too (85.7% “essential”).

The format option reached 80% consensus or higher for a few Delphi items (supplementary Tables 1–4). The format of each item included in the final template was chosen by the writing committee by taking into account the preferences of the majority of the RPs and PPs. Likewise, given the lack of consensus on the description of the CT findings other than CT features of FLD and the suggestion of reporting ancillary findings in round 2, an item “other CT findings” (to be reported as “free text”) was included in the final structured report template.

**Fig. 2:**
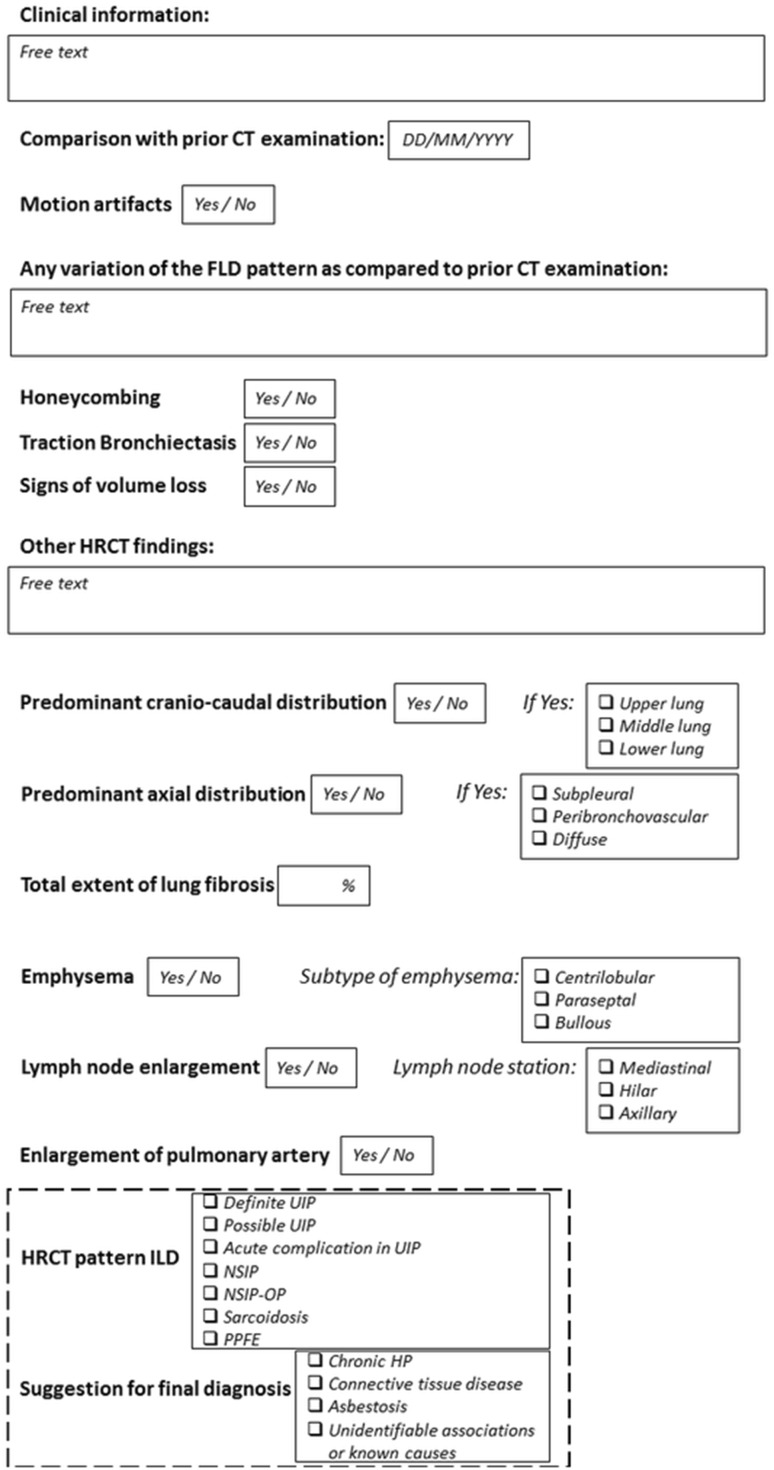
Final structured report template approved by all the study participants

By accounting for the rounds results, a final structured report template was finally developed by the writing committee, circulated and approved among all the participants (Fig. 2).

## Discussion

We applied a rigorous consensus-building technique to identify the key items to be included in a structured report of FLD. In particular, it is the result of joint discussion between thoracic radiologists and pulmonologists. The iterative structure of the Delphi exercise showed a substantial agreement on the items progressively proposed for inclusion by each board of panelists, at subsequent rounds. The proposed template for structured report of FLD comes as the first multidisciplinary example in the field.

The glossary of terms for thoracic imaging as well as the classification documents have both improved the interpretation of the FLD [18–20]. However, there is no official reference for consistent reporting HRCT scan of subjects with FLD. This deficiency may have an impact on medical practice. Noteworthy, standardized structured reporting may reduce the risk of under-reporting important findings of FLD, especially in peripheral centers. Therefore, as for other specific topics, a structured report may well fit the FLD [21].

Ideally, the radiologic report should address both clinical and radiological needs, with a direct and easy format. On the clinical side, there is no official recommendation about the mandatory features of a radiological report, notably about suggesting association between HRCT pattern and cause of DLD. Furthermore, it is not clear whether the radiologic report should include proposals for the management. Both topics should be discussed according to the potential influence on clinical decision making, even in terms of potential medico-legal consequence [22]. This study was indeed driven by the need for practical interaction to improve the usefulness of the basic communicating system within multidisciplinary management of the patient, namely the radiologic report. Therefore, pulmonologists were involved in this study to integrate their needs in a shared structured report, and to rate the relevance of items selected by radiologists.

Interestingly, pulmonologists agreed on the choice of radiologists and suggested to include information on the extent of both FLD and emphysema, which were not rated “essential” by a sufficient proportion of radiologists. Furthermore, pulmonologists proposed an additional item for the conclusions, namely the suggestion of possible cause or association of the FLD, which might be particularly useful to define the clinical choice for subsequent diagnostic work-up or management. For example, the compulsory radiological suggestion of a potential cause of FLD (e.g., asbestosis, connective tissue disease, etc.) rather than the sole HRCT pattern (e.g., UIP or NSIP), might encourage both radiologists to systematically look for important ancillary findings (e.g., pleural plaques, dilated esophagus, etc.) and pulmonologists to seek clinical investigation for the proposed association [23].

The Delphi exercise is one of the strengths of the present study. Indeed, it is a widely recognized method to investigate consensus among experts. Noteworthy, the anonymity of panelists reduces the potential influence of strong opinion leaders on other participants and, thereby, the results of the survey [24]. The specific design of our Delphi survey was meant to first gather the opinion of radiologists, and then to investigate agreement of pulmonologists, especially on topics that are essential to the majority of expert radiologists. This logical sequence was particularly important for technical and medico-legal issues, which may be beyond the radiological “know how” of pulmonologists. Therefore, this approach granted main radiological address of the report with clinically significant adjustment from pulmonologists. The unanimous agreement of pulmonologists on items retained by radiologists suggests that all those items were clinically necessary, but allegedly they were not sufficient.

There are multiple formats for structured report, with no one being absolutely preferable over the other. They range from free text reporting with reference headers to predefined point-and-click options [25]. In our study, the panelists were asked to choose their preferred format for each item. There was poor consensus (threshold for consensus ≥ 80%) on the format for structured report. Both radiologists and pulmonologists did not consider essential the reporting style of HRCT findings, particularly the avoidance of over-description and the disclosure of confidence on interpretation of key HRCT findings (e.g., honeycombing vs reticular abnormalities superimposed to emphysema). Therefore, the format of each item included in the final template was chosen by the writing committee by taking into account the preferences of the majority of the RPs and PPs. We think that a combination of free text and outlined items may be helpful to either systematically disclose key unequivocal HRCT features (e.g., honeycombing) or describe the meaning of individual abnormalities that otherwise would be difficult to capture using a fixed check-list.

Further items included in the final structured report template are worth of discussion. The clinical inquiry should be included in the first rows of the report to facilitate the structured answer to the specific question. Conversely, stating the absence of pertinent clinical history may help convey diagnostic uncertainties [25]. Moreover, the comparison with prior examinations might be prognostically relevant, thus it should be clearly addressed at each HRCT control. The proposed structured report encourages the key discrimination between FLD and non-fibrotic DLD. Notably, the structured description of HRCT signs of FLD allows the list of potential diagnoses of DLD to be narrowed from over a hundred disorders to a handful of diseases [9].

Radiologists did not reach a consensus on the format of preferred visual scoring for emphysema extent. This was probably due to the current lack of clarity about the best method to be applied for that purpose, indeed the visual score is affected by quite an inter-observer variation. Furthermore, the utility of semi-automatic software for emphysema quantitation in routine activity has yet to be clarified, especially for emphysema in FLD [26]. Density masking is the current reference standard for emphysema quantification. This method is not suitable for quantification of patients with emphysema and FLD because it cannot discriminate between low density areas due to emphysema or associated with honeycomb cysts or traction bronchiectasis [27]. On the other hand, the total disease extent by categories would help stratifying patient prognosis, especially in sarcoidosis and systemic sclerosis. This was a topic of debate between radiologists and pulmonologists. In our survey, the radiologists did not reach a consensus on that [13, 14], whilst the pulmonologists preferred to include specific description of disease extent for any FLD as well as emphysema. This request from the pulmonologists is likely related to the need to get additional instruments to improve their interpretation of the patient clinico-functional profile.

The inclusion of air trapping in FLD seemed also concerning for structure report. The predictive value of air trapping in FLD is still under evaluation, with no cut-off extent being recognized. Furthermore, air trapping may be independent from specific association with FLD (e.g., it has been reported in up to 32% of IPF cases) [28]. In a recent study, diffuse air trapping was the source of CT-pathologic discordance in 71.8% in IPF [29].

In the “Conclusions” domain, neither radiologists nor pulmonologists agreed upon suggesting specific diagnostic work-up because it was likely perceived as potentially misleading, allegedly beyond the radiologists knowledge (e.g., radiologists have often limited information about the patient clinical condition and indeed cannot figure out what would be best for him).

This study has some limitations. First, the study panelists were from a single country, for this reason there was a relatively small number of selected expert panelists. The participation of opinion leaders from multiple countries would grant broader sharing and would increase the consistency of the structured report. We foster this National survey will prompt international survey to increase the number of participants, which was likely not sufficiently large to address the study task. The Delphi items were evaluated according to the Italian terminology, nevertheless international scientific references were supplied for informed review. Second, the items were initially selected by the study writing committee, thus resulting in a modified Delphi exercise that could potentially bias the structured report outline. Finally, this study did not aim to assess the impact of the structured report in the diagnosis and management of FLD. This issue will be discussed by forthcoming studies.

In conclusion, this study provides a template for structured report of FLD that features essential items as agreed by thoracic radiologists and their main speaker, the pulmonologists. The multidisciplinary methodology strengthens the structured report utility for daily practice.

## Electronic supplementary material

Below is the link to the electronic supplementary material.

Supplementary material 1 (DOCX 36 kb)

## Abbreviation

DLD: Diffuse lung disease
FLD: Fibrosing lung disease
HRCT: High-resolution computed tomography
NSIP: Non-specific interstitial pneumonia
PPSs: Pulmonology panelists
RPs: Radiology panelists
UIP: Usual interstitial pneumonia
