# Supplementary material for: Structured reporting for fibrosing lung disease: a model shared by radiologist and pulmonologist
Source: Radiol Med. 2017 Dec 11;123(4):245–53. doi: 10.1007/s11547-017-0835-6 (PMC5849634; doi:10.1007/s11547-017-0835-6)
Supplement: Supplementary file 1 — Supplementary material 1 (DOCX 36 kb) [file 11547_2017_835_MOESM1_ESM.docx]

**Supplementary Table 1 – Round 1: items proposed by the writing committee and rated by the radiology panellists (N=42)**

| **Structured Report Items** | **Essential** | **Optional** | **Not relevant** | **missing** | **To be outlined** | **To be given as free text** | **No preferences** |
| --- | --- | --- | --- | --- | --- | --- | --- |
| **Initial Considerations** |  |  |  |  |  |  |  |
| CT protocol details | 69,0% | 11,9% | 16,7% | 2,4% | 57,1% | 11,9% | 31% |
| Available clinical indication | **81,0%** | 16,7% | 2,4% | 0% | 23,8% | 52,4% | 23,8% |
| Comparison with prior CT examinations | **100%** | 0% | 0% | 0% | 42,9% | 33,3% | 23,8% |
| Differences in CT technique with prior examinations | 66,7% | 7,1% | 23,8% | 2,4% | 23,8% | 33,3% | 42,9% |
| Motion artifacts | **92,9%** | 4,8% | 2,4% | 0% | 47,6% | 28,6% | 23,8% |
| **HRCT Findings** |  |  |  |  |  |  |  |
| Comparison of CT findings with prior scan, indicating change of each HRCT finding | **83,3%** | 11,9% | 4,8% | 0,0% | 33,3% | 38,1% | 28,6% |
| Initial assessment of signs lung fibrosis (honeycombing, traction bronchiectasis, signs of volume loss) | **97,6%** | 2,4% | 0,0% | 0,0% | 69,5% | 23,8% | 16,7% |
| Confidence on honeycombing against traction bronchiectasis | 73,8% | 16,7% | 7,1% | 2,4% | 26,2% | 40,5% | 33,3% |
| Avoid description of the absent findings | 57,1% | 28,6% | 14,3% | 0,0% | 28,6% | 31% | 40,4% |
| Description of all CT findings | 45,2% | 26,2% | 28,6% | 0,0% | 16,7% | 31% | 52,4% |
| Description of the most relevant CT findings only (e.g. honeycombing, traction bronchiectasis, signs of volume loss) | 66,7% | 21,4% | 11,9% | 0,0% | 19% | 35,7% | 45,2% |
| Disease distribution on both axial and cranio-caudal planes | **100,0%** | 0,0% | 0,0% | 0,0% | 78,6% | 7,1% | 14,3% |
| Differentiation between macro- and micro-cystic honeycombing | 28,6% | 40,5% | 28,6% | 2,4% | 52,4% | 4,8% | 42,9% |
| Description of the reticular opacities subtypes (e.g. interlobular or interlobular lines) | 59,5% | 28,6% | 9,5% | 2,4% | 66,7% | 7,1% | 26,2% |
| Emphysema subtype including the so-called airspace enlargement with fibrosis | **90,5%** | 4,8% | 2,4% | 2,4% | 69,5% | 19% | 21,4% |
| Quantitation of FLD extent as percentage of the lung volume | 11,9% | 57,1% | 28,6% | 2,4% | 50% | 7,1% | 42,9% |
| Quantitation of FLD extent according to three categories of severity | 69,0% | 26,2% | 4,8% | 0,0% | 66,7% | 2,4% | 31% |
| Quantitation of FLD extent for any disease OR for sarcoidosis and systemic sclerosis only | 11,9% | 57,1% | 28,6% | 2,4% | 50% | 7,1% | 42,9% |
| Quantitation of emphysema extent as percentage of the lung volume | 11,9% | 57,1% | 28,6% | 2,4% | 50% | 7% | 43% |
| Quantitation of emphysema extent according to three categories of severity | 64,3% | 33,3% | 2,4% | 0,0% | 66,7% | 4,8% | 28,6% |
| Report air trapping only when expiratory CT scan is performed | 57,1% | 11,9% | 31,0% | 0,0% | 45,2% | 4,8% | 50% |
| Suggest air trapping also on inspiratory CT scan | 73,8% | 19,0% | 7,1% | 0,0% | 47,6% | 21,4% | 31% |
| Report enlarged pulmonary artery for any disease OR for sarcoidosis and systemic sclerosis only | **87,8%** | 9,8% | 0,0% | 2,4% | 52,4% | 4,8% | 42,9% |
| Report enlarged lymph nodes | **80,5%** | 17,1% | 0,0% | 2,4% | 40,5% | 14,3% | 45,2% |
| **Conclusions** |  |  |  |  |  |  |  |
| CT pattern | **97,6%** | 0,0% | 0,0% | 2,4% | 61,9% | 14,3% | 23,8% |
| Proposal for the subsequent diagnostic test | 40,5% | 54,8% | 0,0% | 5,0% | 45,2% | 26,2% | 28,6% |
| Indication for the timing of CT follow-up | 45,2% | 47,6% | 0,0% | 2,4% | 45,2% | 26,2% | 28,6% |

Note – CT = Computed Tomography; FLD = Fibrosing Lung Disease.

**Supplementary Table 2 – In Round 2 the radiology panelists: A) re-rated those items that did not reach the threshold for consensus in Round 1 and B) classified as ‘to be outlined’ or ‘to be given as free text’ those items that did reach the threshold for consensus on Round 1**

| **Structured Report Items** | **Essential** | **Optional** | **Not relevant** | **missing** | **To be outlined** | **To be given as free text** | **No preferences** |
| --- | --- | --- | --- | --- | --- | --- | --- |
| **Initial Considerations** |  |  |  |  |  |  |  |
| CT protocol details | 51,2% | 46,3% |  | 2,4% | 48,8% | 2,5% | 51,2% |
| Available clinical indication | **Reached threshold for consensus in Round 1** | | | | **56,1%** | 43,9% | 0,0% |
| Comparison with prior CT examinations | **Reached threshold for consensus in Round 1** | | | | **75,6%** | 24,4% | 0,0% |
| Differences in CT technique with prior examinations | 46,3% | 53,7% |  | 0,0% | 36,6% | 9,7% | 56,1% |
| Motion artifacts | **Reached threshold for consensus in Round 1** | | | | **78,0%** | 19,5% | 2,4% |
| **CT Findings** |  |  |  |  |  |  |  |
| Comparison of CT findings with prior scan, indicating change of each CT finding | **Reached threshold for consensus in Round 1** | | | | 41,5% | **58,5%** | 0,0% |
| Initial assessment of signs lung fibrosis (honeycombing, traction bronchiectasis, signs of volume loss) | **Reached threshold for consensus in Round 1** | | | | **85,4%** | 14,6% | 0,0% |
| Confidence on honeycombing against traction bronchiectasis | 78,0% | 17,1% |  | 4,9% | 29,3% | 48,8% | 24,4% |
| Avoid description of the absent findings | 51,2% | 46,3% |  | 2,4% | 39,0% | 12,2% | 51,2% |
| Description of all CT findings | 53,7% | 43,9% |  | 2,4% | 19,5% | 34,1% | 48,8% |
| Description of the most relevant CT findings only (e.g. honeycombing, traction bronchiectasis, signs of volume loss) | 61,0% | 39,0% |  | 0,0% | 41,5% | 19,5% | 41,5% |
| Disease distribution on both axial and cranio-caudal planes | **Reached threshold for consensus in Round 1** | | | | **87,8%** | 12,2% | 0,0% |
| Differentiation between macro- and micro-cystic honeycombing | 24,4% | 73,2% |  | 2,4% | 24,4% | 0,0% | 78,1% |
| Description of the reticular opacities subtypes (e.g. interlobular or interlobular lines) | 58,5% | 39,0% |  | 2,4% | 46,3% | 12,2% | 43,9% |
| Emphysema subtype including the so-called airspace enlargement with fibrosis | **Reached threshold for consensus in Round 1** | | | | **90,2%** | 9,8% | 0,0% |
| Quantitation of FLD extent as percentage of the lung volume | 46,3% | 51,2% |  | 2,4% | 78,1% | 14,6% | 9,7% |
| Quantitation of FLD extent according to three categories of severity | 65,9% | 31,7% |  | 2,4% | 53,7% | 12,2% | 36,6% |
| Quantitation of FLD extent for any disease OR for sarcoidosis and systemic sclerosis only | 46,3% | 51,2% |  | 2,4% | 21,9% | 2,5% | 78,1% |
| Quantitation of emphysema extent as percentage of the lung volume | 31,7% | 68,3% |  | 0,0% | 26,8% | 4,9% | 70,7% |
| Quantitation of emphysema extent according to three categories of severity | 65,9% | 34,1% |  | 0,0% | 58,5% | 7,3% | 36,6% |
| Report air trapping only when expiratory CT scan is performed | 51,2% | 46,3% |  | 2,4% | 43,9% | 7,3% | 51,2% |
| Suggest air trapping also on inspiratory CT scan | 78,0% | 22,0% |  | 0,0% | 53,7% | 24,4% | 24,4% |
| Report enlarged pulmonary artery for any disease OR for sarcoidosis and systemic sclerosis only | **Reached threshold for consensus in Round 1** | | | | **66,7%** | 23,8% | 9,5% |
| Report enlarged lymph nodes | **Reached threshold for consensus in Round 1** | | | | **61,9%** | 28,9% | 9,5% |
| **Conclusions** |  |  |  |  |  |  |  |
| CT pattern | **Reached threshold for consensus in Round 1** | | | | **82,9%** | 17,1% | 0,0% |
| Proposal for the subsequent diagnostic test | 46,3% | 53,7% |  | 0,0% | 39,0% | 7,3% | 56,1% |
| Indication for the timing of CT follow-up | 46,3% | 51,2% |  | 2,4% | 34,1% | 12,2% | 56,1% |

Note – CT = Computed Tomography; FLD = Fibrosing Lung Disease.

**Supplementary Table 3 – In Round 3 the pulmonologists panelists: A) re-rated those items that did not reach the threshold for consensus in Round 1 and B) classified as ‘to be outlined’ or ‘to be given as free text’ those items that did reach the threshold for consensus on Round 1**

| **Structured Report Items** | **Essential** | **Optional** | **Not relevant** | **missing** | **To be outlined** | **To be given as free text** | **No preferences** |
| --- | --- | --- | --- | --- | --- | --- | --- |
| **Initial Considerations** |  |  |  |  |  |  |  |
| CT protocol details | 33,3% | 66,7% |  |  | 16,7% | 16,7% | 66,7% |
| Available clinical indication | **AGREE WITH RP CONSENSUS** | | | | | | |
| Comparison with prior CT examinations | **AGREE WITH RP CONSENSUS** | | | | | | |
| Differences in CT technique with prior examinations | 41,7% | 58,3% |  |  | 25% | 25% | 50% |
| Motion artifacts | **AGREE WITH RP CONSENSUS** | | | | | | |
| **HRCT Findings** |  |  |  |  |  |  |  |
| Comparison of CT findings with prior scan, indicating change of each HRCT finding | **AGREE WITH RP CONSENSUS** | | | | | | |
| Initial assessment of signs lung fibrosis (honeycombing, traction bronchiectasis, signs of volume loss) | **AGREE WITH RP CONSENSUS** | | | | | | |
| Confidence on honeycombing against traction bronchiectasis | 33,3% | 66,7% |  |  | 25% | 25% | 50% |
| Avoid description of the absent findings | 41,7% | 58,3% |  |  | 25% | 25% | 50% |
| Description of all CT findings | 41,7% | 58,3% |  |  | 25% | 8,3% | 66,7% |
| Description of the most relevant CT findings only (e.g. honeycombing, traction bronchiectasis, signs of volume loss) | 16,2% | 83,3% |  |  | 8,3% | 8,3% | 83,3% |
| Disease distribution on both axial and cranio-caudal planes | **AGREE WITH RP CONSENSUS** | | | | | | |
| Differentiation between macro- and micro-cystic honeycombing | 8,3% | 91,6% |  |  | 8,3% | 0,0% | 91,7% |
| Description of the reticular opacities subtypes (e.g. interlobular or interlobular lines) | 25% | 75% |  |  | 25% | 0,0% | 75,0% |
| Emphysema subtype including the so-called airspace enlargement with fibrosis | **AGREE WITH RP CONSENSUS** | | | | | | |
| Quantitation of FLD extent as percentage of the lung volume | 100 | 0% |  |  | 0,0% | 0,0% | 100% |
| Quantitation of FLD extent according to three categories of severity | 25% | 75% |  |  | 16,7% | 8,3% | 75,0% |
| Quantitation of FLD extent for any disease OR for sarcoidosis and systemic sclerosis only | 25% | 75% |  |  | 33,3% | 0,0% | 66,7% |
| Quantitation of emphysema extent as percentage of the lung volume | 25% | 75% |  |  | 16,7% | 8,3% | 75,0% |
| Quantitation of emphysema extent according to three categories of severity | 100% | 0% |  |  | 16,7% | 16,7% | 66,7% |
| Report air trapping only when expiratory CT scan is performed | 41,7% | 58,3% |  |  | 16,7% | 33,3% | 50% |
| Suggest air trapping also on inspiratory CT scan | 41,7% | 58,3% |  |  | 25% | 25% | 50% |
| Report enlarged pulmonary artery for any disease OR for sarcoidosis and systemic sclerosis only | **AGREE WITH RP CONSENSUS** | | | | | | |
| Report enlarged lymph nodes | **AGREE WITH RP CONSENSUS** | | | | | | |
| Other ancillary findings | **AGREE WITH RP CONSENSUS** | | | | | | |
| **Conclusions** |  |  |  |  |  |  |  |
| CT pattern | **AGREE WITH RP CONSENSUS** | | | | | | |
| Proposal for the subsequent diagnostic test | 33,3% | 66,7% |  |  | 16,7% | 16,7% | 66,7% |
| Indication for the timing of HRCT follow-up | 16,2% | 83,3% |  |  | 0.0% | 16,7% | 83,3% |

Note – CT = Computed Tomography; FLD = Fibrosing Lung Disease.

**Supplementary Table 4 – Item proposed by the pulmonology panelists for the radiology panelists**

| **OPTION A + B (Essential for 85,7%)*** | |
| --- | --- |
| **OPTION A (Essential for 14.3%)** | **OPTION B (to be considered only in association with option A)** |
| UIP DEFINITE | UNIDENTIFIABLE ASSOCIATIONS OR KNOWN CAUSES |
| UIP POSSIBLE | CONNECTIVE TISSUE DISEASE |
| UIP POSSIBLE OR DEFINITE WITH SIGNS OF ACUTE COMPLICATIONS (TO BE SPECIFIED) | ASBESTOSIS |
| NSIP | CHRONIC HYPERSENSITIVITY PNEUMONITIS |
| NSIP-OP |  |
| FIBROSING SARCOIDOSIS |  |
| PPFE |  |
| UNCLASSIFIABLE |  |

Note – NSIP = Non-Specific Interstitial Pneumonia; OP = organizing pneumonia; PPFE = Pleuroparenchymal Fibroelastosis; UIP = Usual Interstitial Pneumonia

*The majority (85.7%) of the RPs
